# Take home lessons from studies of related proteins

**DOI:** 10.1016/j.sbi.2012.11.009

**Published:** 2013-02

**Authors:** Adrian A Nickson, Beth G Wensley, Jane Clarke

**Affiliations:** Department of Chemistry, University of Cambridge, Lensfield Rd, Cambridge CB2 1EW, UK

## Abstract

The ‘Fold Approach’ involves a detailed analysis of the folding of several topologically, structurally and/or evolutionarily related proteins. Such studies can reveal determinants of the folding mechanism beyond the gross topology, and can dissect the residues required for folding from those required for stability or function. While this approach has not yet matured to the point where we can predict the native conformation of any polypeptide chain *in silico*, it has been able to highlight, amongst others, the specific residues that are responsible for nucleation, pathway malleability, kinetic intermediates, chain knotting, internal friction and Paracelsus switches. Some of the most interesting discoveries have resulted from the attempt to explain differences between homologues.

## Highlights

► We review the recent advances made from the study of related proteins. ► We relate pathway malleability to the balance between foldons and helical propensity. ► We speculate why different topologies respond differently to mutation. ► We discuss the role of kinetic intermediates in folding pathways. ► We explain why it is important to study several members from each protein fold.

**Current Opinion in Structural Biology** 2013, **23**:66–74

This review comes from a themed issue on **Folding and binding**

Edited by **Jayant Udgaonkar** and **Susan Marqusee**

For a complete overview see the Issue and the Editorial

Available online 20th December 2012

0959-440X/$ – see front matter, © 2012 Elsevier Ltd. All rights reserved.

**http://dx.doi.org/10.1016/j.sbi.2012.11.009**

## Introduction

In the fifty years since the protein-folding field was first established, there have been thousands of papers detailing the thermodynamic or kinetic characterization of hundreds of different proteins. One particularly useful approach is ‘The Fold Approach’ [1], which involves a detailed analysis of the folding of several topologically, structurally and/or evolutionarily related proteins in order to discern patterns and trends in folding (stability, pathways and mechanisms).

In this manuscript, we describe a number of studies that highlight how comparisons within and between related protein families have affected our understanding of protein folding. This article builds on our recent review [2^•^] incorporating significant results from the last few years. Here, we focus on the folding of isolated domains and do not discuss multidomain proteins, misfolding or aggregation.

## The malleability of protein folding pathways

### A unifying folding mechanism

In the early days of the ‘protein-folding problem’, three competing mechanisms were proposed that described how a polypeptide chain might fold to the native state: nucleation [3], hydrophobic-collapse [4] and diffusion-collision (framework) [5]. However, an early *Φ*-value analysis of the small protein chymotrypsin inhibitor 2 (CI2) demonstrated that none of these mechanisms was appropriate, since secondary and tertiary structure formed concomitantly [6]. Thus the nucleation-condensation mechanism was introduced [7], in which long-range contacts set up the initial topology of the protein (incurring a substantial entropic loss with minimal enthalpic gain), followed by a rapid collapse to the native state (with minimal entropic loss but substantial enthalpic gain). Under these conditions, the transition state is usually an expanded form of the native state [8], which helps to explain the strong correlation between native topological complexity (Contact Order) and folding rates, as noted by Plaxco and Baker in the late 1990s [9].

Although the nucleation-condensation mechanism is observed to be widely applicable, several proteins have been shown to fold in a more hierarchical manner. In particular, the engrailed homeodomain (En-HD) was seen to fold via a classical framework mechanism [10]. To investigate whether this result was owing to the simple architecture of the protein, Fersht and co-workers studied four other members of the homeodomain-like superfamily: c-Myb, hRAP1, Pit1 and hTRF1. They observed a slide in mechanism a slide from hTRF1 (pure nucleation-condensation) to En-HD (pure framework) through c-Myb, hRAP1 and Pit1 (mixed mechanisms), which correlated with the innate secondary structural propensity of each domain [11,12^•^]. The authors used this result to conclude that nucleation-condensation and diffusion-collision are thus “different manifestations of a common unifying mechanism” for protein folding. This variation is not unique, and a continuum of mechanisms has also been seen for different members of the PSBD superfamily, where it is again linked to secondary structural propensity [13].

### The foldon concept

Further reconciliations between apparently different folding pathways have also been proposed using the concept of ‘foldons’. This term was initially used to describe the C-terminal domain of bacteriophage T4 fibritin [14], but was quickly adopted by Wolynes and co-workers to describe independently folding units of a protein chain [15]. Although originally referring solely to contiguous regions of polypeptide sequence, Englander [16] and Oliveberg [17,18] redefined the term ‘foldon’ to describe any kinetically competent submotif within a protein (i.e. any subset of residues that can fold cooperatively to a defined structural state).

Perhaps the most successful application of the foldon hypothesis comes from studies of the ferredoxin-like family of proteins including U1A and the small ribosomal protein S6 from Thermus thermophilus (S6_T_). Here, Oliveberg and co-workers observed that, while the wild-type S6_T_ protein folded through a globally diffuse transition state that typified nucleation-condensation, a circular permutant (with conjoined wild-type termini and a different backbone cleavage site) exhibited an extremely polarized transition state [19]. Moreover, two alternate circular permutants demonstrated that entropy mutations could be used to shift the position of the nucleus within the topology of the S6_T_ protein [20]. This finding was particularly interesting, since it reconciled the folding of S6_T_ and U1A with that of S6_A_ and ADA2h: two other homologous ferredoxin-like proteins that appeared to fold through a different pathway (although still by nucleation condensation). Oliveberg explained these results by suggesting that all ferredoxin-like proteins comprise two overlapping foldons, but that the specific folding pathway is determined by the primary sequence of each domain [18].

It is, perhaps, easiest to compare these foldons to tandem repeat proteins. In these proteins, each repeat is unstable in isolation – and yet each repeat has a defined native structure to which it will fold [21,22^•^]. Interactions between these repeats can provide sufficient stabilization to produce a globally stable native state, and a cooperatively folding protein [23]. In the same way, isolated foldons are unstable – but the combination of several foldons will lead to a stable, structured protein domain. In the ankyrin repeat protein myotrophin, it is the C-terminal repeat that is most stable (least unstable) in isolation, and hence folding begins in this region of the protein. However, when this repeat is destabilized by mutation, it is now the N-terminal repeat that is most stable, and the protein will fold from the opposite end over a different pathway [24], similar to that of Internalin B [25]. A similar rerouting of the folding pathway has also been achieved by mutations in the Notch ankyrin domain [26]. In an analogous manner, the folding of the ferredoxin-like proteins is controlled by which of the two component foldons is the most stable (least unstable), hence the differences in transition state structure between U1A/S6_T_ and S6_A_/ADA2h [18].

### How do folding pathways respond to sequence changes?

**Figure 1:**
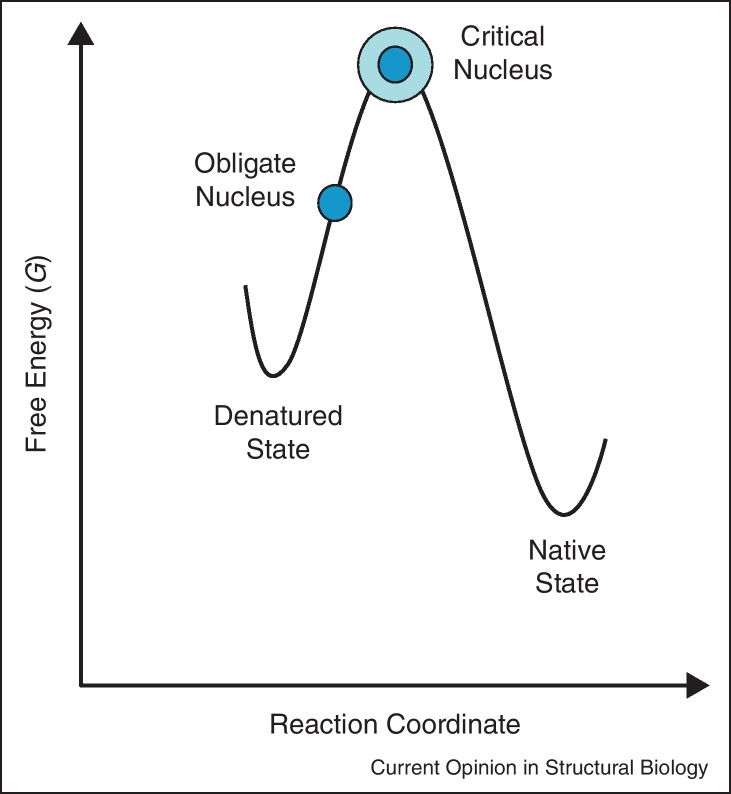
Folding by nucleation condensation: the key elements of the folding nucleus. The folding nucleus can be subdivided into the obligate nucleus (dark blue) and the critical nucleus (cyan) [27,28]. The obligate nucleus brings together those elements of secondary structure that are required to set up the native protein topology. Interactions between what have been called the ‘key residues’ [88] form early, and are associated with a high entropy cost and little enthalpic gain. The critical nucleus forms a shell around the obligate nucleus, and provides sufficient extra interactions to turn the free-energy profile downhill, (lower entropic cost, larger enthalpic gain). These interactions are more plastic, and only a subset of these interactions may be required to complete the folding nucleus.

**Figure 2:**
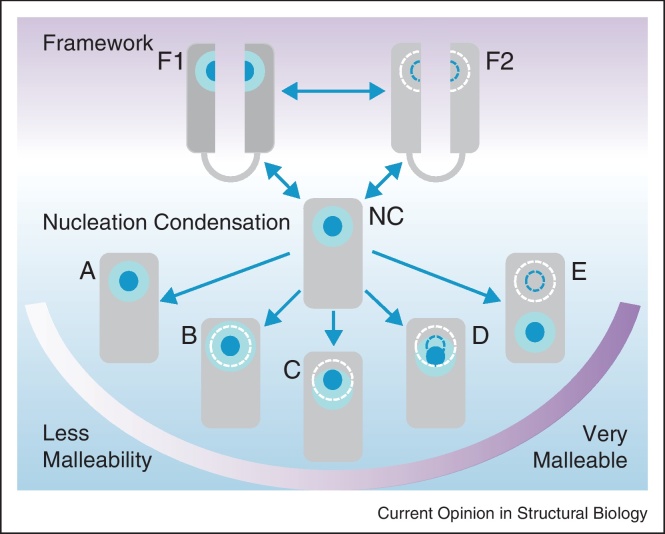
How folding mechanisms or pathways might change when the sequence of a protein changes. Top: Protein folding has been described as occurring by a sliding mechanism between a framework mechanism, F (5), and nucleation condensation, NC [7]. (F1) If the secondary structure (helical) propensity of the protein is high (dark grey) then secondary structure formation may precede the formation of a tertiary folding nucleus and the protein folds through the framework mechanism. If the secondary structure weakens then a nucleation-condensation mechanism may become more favourable. (F2) If the secondary structure propensity is weak (light grey), but there is no strong nucleus, the protein may still fold by a framework-like, diffusion-collision mechanisms, where folding proceeds through collision of partly formed secondary structure elements. Changes in sequence may lead to stronger, earlier formation of secondary structure, or a move to nucleation condensation. Bottom: Within nucleation condensation (NC) mechanisms there may be shifts in the folding nucleus. The malleability of a protein-folding pathway is determined by its component foldons and by redundancy in the nucleating residues. The obligate nucleus is shown in blue and the critical nucleus is shown in cyan. **(a)** Where a protein contains only one potential set of nucleating residues, the folding pathway is robust. Such proteins can be described as ‘ideal’ two state folders, and exhibit V-shaped chevron plots with a single free-energy barrier. Mutation of the nucleating residues will not change the structure of the transition state, but may result in a protein that cannot fold. (**b** and **c**) If the obligate nucleus is surrounded by many favourable interactions, then a detrimental mutation within the critical nucleus can lead to the recruitment of other interactions to compensate. This will result either in expansion of the critical nucleus, **b**, or a shift in the position of the nucleus, **c**. Such mutations can lead to Hammond effects. (**d** and **e**) If a protein can use degenerate residues to set up its native state topology, then mutations within the obligate nucleus can lead to minor shifts in both the obligate nucleus and the critical nucleus; however, if the topology provides alternate foldons, then disruption of the obligate nucleus may result in a complete shift in the position of the folding nucleus. These latter shifts are often linked to anti-Hammond behaviour. Alternatively, in the absence of an alternative set of nucleating residues, destruction of the folding nucleus may lead to a protein that can only fold when transient secondary structure is stabilized by long-range tertiary interactions (F2). Such a protein would be said to fold through the diffusion-collision mechanism.

Both experiment [27] and theory [28] suggest that the protein-folding nucleus can be subdivided into two distinct sections (Figure 1). The *obligate nucleus* comprises those few interactions that commit the polypeptide chain to fold to the correct native state topology. Such residues pack early, (with high *Φ*-values), and incur a substantial entropy cost with little enthalpic gain. They are surrounded by the *critical nucleus*, which is a shell of additional interactions that are necessary to turn the free-energy profile downhill (i.e. additional interactions that are accumulated up to the global transition state). These interactions are more plastic, and each folding event may use a different subset of residues within the critical nucleus to effect a barrier crossing. The foldon idea can be combined with that of the obligate and critical folding nucleus to explain the many types of pathway malleability: this is described in Figure 2, and exemplified by members of the immunoglobulin-like (Ig-like) fold.

When considering the folding of related proteins, perhaps the most thoroughly studied fold is that of the Ig-like domains. These all-β proteins have a complex Greek-key architecture, and are extremely common in eukaryotes with over 40 000 distinct domains identified to date [29]. They were chosen for study because, despite their complex topology, there is low sequence identity within each superfamily – and virtually no sequence identity between different superfamilies. Early studies on fibronectin type III (fnIII) domains (TNfn3 and FNfn10) revealed the presence of four key hydrophobic residues in the B, C, E and F strands that constituted the obligate nucleus: interactions of these residues was necessary, but sufficient, to set up the correct topology of the protein [30–32]. Interestingly, the size of the critical nucleus was very different in these two proteins – it is far more extensive in FNfn10 than in TNfn3 (Figure 2B). Moreover, in FNfn10, a few mutations resulted in a small change in the unfolding *m*-value that could indicate a shift in the critical nucleus (Figure 2C). Most importantly, the obligate nucleus of the evolutionarily unrelated Ig domain titin I27 comprised residues that were structurally equivalent to those in the fnIII domains [33]. Thus, these proteins share an obligate nucleus, which is required to set up the correct topology of these complex Greek-key domains and allow folding to proceed. Indeed, the hydrophobic residues of this obligate nucleus were so well conserved that a search of the Protein Data Bank (PDB) was undertaken to find an Ig-like domain that did not contain this nucleation motif. The resultant domain, CAfn2, was subject to a detailed *Φ*-value analysis that produced a gratifying result: the folding nucleus had simply ‘slipped’ down the core to use an adjacent pair of hydrophobic residues [34] – both the obligate and critical nuclei have moved in response to sequence changes (Figure 2D).

A final surprise in this analysis of pathway malleability in Ig-like domains came from a more detailed analysis of I27. This domain exhibited unusual anti-Hammond behaviour at high concentrations of denaturant and upon mutation. These data were used to infer the presence of an alternate folding pathway that nucleated at the E–F loop – both the critical *and* the obligate nucleus have moved entirely (Figure 2E) [35]. Thus we find that Ig-like domains contain at least two *potential* nucleation motifs, with one foldon comprising the B, C, E and F strands and one foldon centred on the E–F loop. Note that we are not implying that every immunoglobulin-like domain can display all types of pathway malleability, merely that the topology of the immunoglobulin fold allows for each. We speculate that this robustness to sequence changes might account for the success of this fold in Nature.

Are all protein folds as malleable? Using a stringent definition for transition state inflexibility, no shift in the position or size of the folding nucleus, the classic two state folder CI2 and the small three-helix bundle BdpA are the only domains for which no experimental perturbation has resulted in an altered transition state structure (Figure 2A). In the case of CI2, this inflexibility extends to point mutation, circularization, circular permutation [36] and even bisection [37], and it appears that this protein really does have only one energetically accessible nucleation motif. However, since no other members of this fold have been studied, it is not yet known if this is a general feature of this protein topology. The BdpA protein has been less ruthlessly perturbed and, while the transition state is not affected by point mutation or by temperature [38,39], a more serious structural perturbation may yet have an effect. An interesting case is demonstrated by the LysM domain, which shows an identical pattern of *Φ*-values after circularization [40], albeit with a global decrease in magnitude. A detailed Eyring analysis suggests that the lower entropy cost of transition state formation is compensated for by a lower enthalpy of contacts: the protein still folds through the same pathway, with a structurally identical but spatially expanded nucleus (Figure 2B).

The apparent malleability of the transition state ensemble can be strongly dependent on the imposed perturbation, as demonstrated by the β-sandwich domain α-spectrin SH3. The wild-type transition state is formed from the packing of two out of the three native state β-hairpins (RT loop and distal loop). A circular permutant that cleaved the RT loop resulted in an unchanged folding pathway, (Figure 2B), but an alternate permutant that cut the distal loop resulted in a completely different transition state structure involving the n-Src loop and the WT termini (Figure 2E) [41]. Other large-scale shifts in the obligate nucleus are not uncommon, especially where the folds exhibit symmetry. The symmetrical, ubiquitin-like Protein G, which comprises a central helix packing on two terminal hairpins, is a good example of such a large change. The wild type protein nucleates using the C-terminal hairpin and helix, as determined by *Φ*-value analysis [42]. However, a computationally redesigned version of the protein was successfully engineered to fold via the N-terminal hairpin [43], with a transition state reminiscent of the homologous Protein L [44]. In both of these cases, SH3 and ubiquitin-like domains, the protein topology provides at least two foldons, either of which is able to nucleate under the right conditions. As with the S6 proteins, these foldons are overlapping.

## The role of intermediates in folding

As mentioned previously, the engrailed homeodomain has been shown to fold through a framework mechanism [11]. In fact, the secondary structural propensity of En-HD is so high that individual helices are stable in isolation (Figure 2, F1). This leads to three-state folding behaviour where kinetic intermediates accumulate. Reducing the secondary structural propensity results in a domain where no helix is stable in isolation. Now, the transiently formed helices are only stabilized once they have accumulated sufficient long-range interactions, and this interdependency results in global folding cooperativity, as seen with c-Myb. This behaviour is shown in Figure 2 as the slide from framework (F1) to nucleation condensation (NC). Nevertheless, c-Myb can be specifically mutated to increase the helical propensity, and convert the folding kinetics to three-state [45]. A similar effect is seen with the immunity proteins, Im7 and Im9 [46,47], which share a common transition state structure despite the fact that Im9 folds in a two-state manner (no independently stable submotifs) while Im7 exhibits three-state kinetics (with at least one independently stable submotif). By stabilizing the nucleating foldon, Im9 was rationally engineered to fold through a kinetic intermediate, while retaining the transition state structure of the homologous Im7 domain [48]. This switch does not always require substantial redesign, as shown by some elegant studies of RNase H, which demonstrated that a single point mutation (Ile to Asp) is sufficient to remove an on-pathway folding intermediate and thus energetically couples the two subdomains of the protein [49^•^,50]. Transiently populated intermediates have also been introduced into, or removed from, the lipocalins [51,52], the immunoglobulin-like proteins [53] and the cytochromes [54] without altering the transition state structure. Taken together, these studies are proof that a folding pathway cannot be solely defined by its kinetic intermediates.

**Figure 3:**
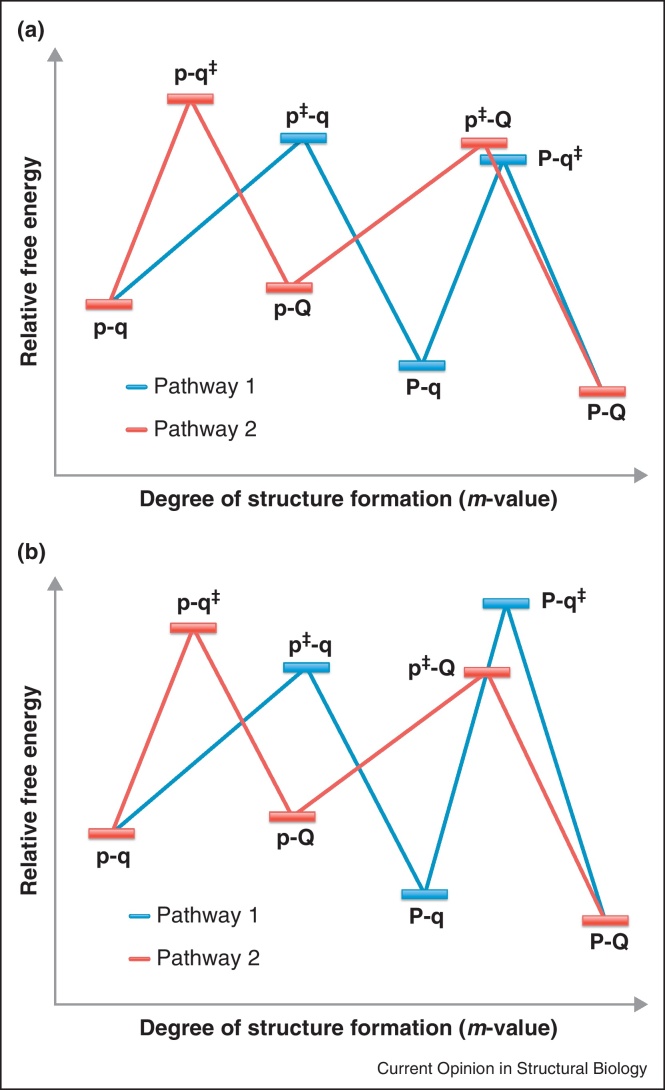
A protein with more than one foldon has access to multiple folding pathways and may exhibit both on-pathway and off-pathway intermediates. Lowercase letters denote unstructured foldons (p, q) and uppercase letters denote structured foldons (P, Q). The double dagger (‡) denotes the foldon that is (un)folding at each transition state. **(a)** Both the PDZ domains and lysozyme have been shown to fold through a triangular folding scheme under certain experimental conditions. This can be explained by considering a protein with two component foldons (p, q) either of which can fold first. Importantly, one foldon is stable in isolation (P) but the other is unstable in isolation (q). In the blue pathway, the second energy barrier (q folding) is larger than the first energy barrier (p folding) and therefore an on-pathway intermediate accumulates. In the red pathway, the intermediate (p-Q) is unstable and folding is two-state. If the highest energy transition states on each pathway are close in energy, (here: p^‡^-q and p-q^‡^), there is significant flux over both folding routes (about 3:2 blue:red for the PDZ domains, and 4:1 for the lysozyme domain). **(b)** Under alternative experimental conditions, formation of one foldon may actually hinder the folding of the second foldon: the energy barrier p-q to p-q^‡^ is lower than the energy barrier P-q to P-q^‡^. Although the majority of the denatured proteins (p-q) fold along the blue pathway to the intermediate (P-q), it is actually less energetically costly for this intermediate to unfold and follow the alternate red pathway than it is for the protein to fold directly from the intermediate to the native state. In this case, the intermediate would appear to be off-pathway – despite the fact that it is possible for the intermediate to fold directly to the native state. This may be the case for the PDZ domain when the temperature is dropped from 37 °C to 25 °C. The intermediate P-q is the same in both cases, but the relative heights of the four energy barriers determine whether or not it is on-pathway **(a)** or off-pathway **(b)**.

A slightly different result came from studies on five homologous members of the PDZ domain-like fold. In each case, the protein was shown to fold over two sequential transition states with a high-energy intermediate. As with Im9, this intermediate was deliberately stabilized, and the resulting domain did indeed fold with three-state kinetics [55]; however, the stabilized intermediate was subsequently shown to be off-pathway [56^••^]. Moreover, a human PDZ domain was found to fold through an intermediate that was either on- or off-pathway, depending on the solution conditions [56^••^]. This is an extremely interesting example where one of the component foldons has mutated so as to be the most stable species under certain solution conditions, as shown by the presence of an equilibrium intermediate. We infer that the PDZ domain contains at least two nucleation competent motifs within its structure. If the protein nucleates using the first (stable) foldon, then the second energy barrier is larger than the first and an intermediate accumulates (Figure 3A). If, however, the protein nucleates using the second (unstable) foldon, then the second energy barrier is smaller than the first and the whole folding process is cooperative. Under certain experimental conditions, it is easier for the intermediate to fully unfold and follow the alternate nucleation pathway than it is for the intermediate to progress directly to the native state (Figure 3B). In these cases, the intermediate appears to be off-pathway. The PDZ behaviour was modeled on that of lysozyme, which contains a stable α-domain, an unstable β-domain, and folds with a ‘triangular’ scheme of two parallel pathways, only one of which exhibits a kinetic intermediate [57]. Alternative folding pathways and kinetic traps have also been observed, and analysed, for homologous members of the flavodoxin-like fold [58,59^•^], the β-trefoil family [60,61] and the caspase recruitment domains [62], amongst others.

## Comparisons between folds

Both spectrin domains and homeodomains are three-helix bundle proteins. Three spectrin domains have been investigated in detail, (R15, R16 and R17), all from chicken brain α-spectrin. As seen for the homeodomains, there is no common folding mechanism, with R16 (and R17) folding by the collision of partly pre-formed helices [63,64], while R15 folds by classical nucleation-condensation [65]. In the spectrin case, however, it is not increased helical propensity in R16 that favours the framework-like mechanism: rather, it is the lack of a competent folding nucleus (Figure 2, F2). Addition of a nucleus results in a change in the folding mechanism *from* framework *towards* nucleation condensation, as shown in Figure 2 with a slide from F2 to NC [66^••^,67^•^]. Interestingly, in contrast to the homeodomains where the framework mechanism leads to faster folding, in spectrin it is the proteins that fold by nucleation condensation that fold faster. This difference is probably related to the difference in size of these two folds. The helices in spectrin are long (8–10 turns per helix) unlike the short 2–3 turn helices in the homeodomains. We have speculated that there is a frustrated search for the correct docking of the helices in the spectrin domains, manifested as ‘internal friction’, that explains this observation [66^••^,68,69]. Remarkably, it has not been possible to alter the folding pathway of R15, either to move towards a framework-like mechanism, or to induce a change in the position of the nucleus: radical destabilization of the folding nucleus in R15, which causes significantly slower folding and unfolding, still results in a protein with *Φ*-values that are identical to the wild-type protein (unpublished data). This protein therefore shows no signs of pathway malleability (Figure 2A), unlike its homologues R16 and R17.

## Combining experiment and computational studies

### Knotted proteins

One of the more surprising results in recent years is the finding that knotted proteins are able to fold spontaneously, without chaperones or enzymatic help, to the native knotted state. Mallam and Jackson investigated two members of the α/β knot family and observed that both YbeA and YibK folded with similar rates and through comparable kinetic pathways, from knotted denatured states [70]. In an elegant recent follow-up study [71^•^], the authors followed the folding of these proteins in a cell-free translation system and demonstrated that the newly synthesized proteins have to knot before they can fold – a rate limiting process that is accelerated by chaperonins. Nevertheless, this knotting process must be controlled by the primary sequence of the protein and thus it is very interesting to investigate homologous proteins where some are knotted and some are not. Faccioli and co-workers used coarse-grained protein models to study the folding of the natively-knotted N-acetylornithine carbamoyltransferase (AOTCase) and a homologous unknotted ornithine carbamoyltransferase (OTCase). They found that, when non-native interactions were ignored, neither protein was able to form a trefoil knot. By contrast, when non-native interactions were added to the model, the AOTCase was able to spontaneously knot in a substantial proportion of the simulations [72^••^]. This kind of study is particularly useful, since it can be used to highlight important folding contacts that cannot be deduced from the native, denatured or transition states. In this case, the simulations predict contacts that can be added/removed *in vitro* to make a knotted form of OTCase or a non-knotted mutant of AOTCase.

### Nearly the same sequence but a different fold

As a contrast to the fold approach, several groups have been working towards designing proteins with highly similar amino acid sequences, but which cooperatively fold to different native state topologies. This quest, known as the Paracelsus Challenge, was first achieved in 1997 when Reagan and co-workers designed two proteins that were more than 50% identical yet adopted different native folds (ROP-like and ubiquitin-like) [73]. This design was surpassed in 2005, and again in 2008, when Bryan and co-workers developed two polypeptide chains that are 88% identical and yet adopt very different tertiary structures [74]. These proteins have been studied both by experiment and computationally, and the conclusion is that the final native topology is determined by the structure of the denatured state and the very earliest folding events [75,76^••^]. In the case of the G_X_88 proteins, the early development of a β-hairpin in one sequence prevents α-helical formation in that region, and leads to the ubiquitin-like fold [75,76^••^]. The alternate sequence retains significant helical structure in the denatured state, which leads to the all-α helical bundle. Residual structure in the denatured state has also recently been shown to be important for the folding of the ribonuclease domains [77^•^] and the SUMO proteins [78].

In a more recent extensive study of the designed system Gianni and co-workers have shown that G_A_88 folds using a robust transition state to a three helical bundle, while G_B_88 folds over a very malleable energy landscape to a ubiquitin-like (mostly β-sheet) topology [79]. This malleability is assigned to the presence of multiple, competing foldons. In contrast to most natural proteins, where the component foldons work in unison to provide a cooperatively folded protein, the Gx88 designed proteins provide an example where two structurally overlapping foldons work in opposition. By fine-tuning the energy cost of each nucleating foldon, the overall topology of the whole protein can be adjusted. This result should be directly applicable to the study of aggregation-prone polypeptides, where minimal perturbations in structure and/or solution conditions are able to change the resulting topology of the folded state from native to the universal cross-β amyloid structure.

## Summary

What is clear from many of these studies is that researchers should be wary of characterising the folding of a particular protein topology based on a single member of the fold. While it may be informative to study a wide cross-section of the proteome [80], gross comparisons between different folds are unable to inform as to how and why a polypeptide chain folds to its specific native state. These answers mostly come from more intricate studies, looking for differences in the folding of closely related proteins (the so-called ‘Fold Approach’). For example, such studies have taught us that a folding pathway should not be defined by its kinetic intermediates, since these species can easily be introduced into, or removed from, the energy landscape (e.g. En-HD/c-Myb, Im7/Im9, PDZ). In addition, while some proteins appear to be very restricted in their response to mutation (CI2, LysM), other folds exhibit a high degree of pathway malleability. This latter group includes the immunoglobulin-like domains, which are able to change their folding nucleus in response to deletions in the hydrophobic core [34], changes in solvent conditions [35], and even under mechanical stress [81,82]. This plasticity in the energy landscape may confer an evolutionary advantage over more restricted folds, and may explain why the topologically complex Ig-like domains are so prevalent when compared to more simple folds: changes in sequence that are required for functional reasons can be easily compensated for by a shift in the folding nucleus. It is also observed that symmetric proteins, such as the ubiquitin-like domains [42,44], show more pathway malleability than similarly sized asymmetric proteins – presumably owing to the comparable entropic cost of topologically symmetric foldons [83,84].

The idea that protein domains comprise several foldons (individually cooperative submotifs) is particularly appealing, since it is able to simplify the folding of complex topologies by introducing the concept of a ‘funnel of funnels’ [85]. This would also have the advantage that *de novo* proteins could be systematically built using a toolbox of smaller components. Indeed, Baker and co-workers recently emphasized that it is easy to rationally stabilize the native state of a protein, but it is much harder to disfavour the plethora of non-native states that are also possible. Their phenomenal success in designing five new stable, monomeric proteins from scratch was based on the structural overlap of several defined motifs with a known topological bias, specifically chosen to favour funnel-shaped energy landscapes [86^••^]. While it is certainly true that the ferrodoxin-like proteins comprise two overlapping foldons, whether or not this is a general feature of all complex protein folds remains to be seen. Nevertheless, one interesting observation is that the size of the dominant foldon may be related to topological complexity. The spectrin repeats [67] and homeodomain-like bundles [12^•^] each nucleate using two of the three helices; the LysM domain [40], ferrodoxin-like proteins and ubiquitin-like domains [18] appear to use a three component foldon; finally, the complex Greek-key immunoglobulin-like domains [33] and Death Domains [87] use a four-component foldon. While this scaling is not necessary for the protein to fold correctly, (for example, the Ig-like domains can fold using the simple E–F loop motif [35]), it may be an evolutionary method to ensure that the protein folds cooperatively and avoids misfolding or aggregation.

In summary, the ‘fold approach’ has contributed significantly to our understanding of the fundamental principles underpinning the efficient folding of evolved proteins on relatively smooth, funnel-like energy landscapes. Furthermore, such studies allow insight into the design of new proteins that can fold efficiently, on funnel-shaped energy landscapes.

## References and recommended reading

Papers of particular interest, published within the period of review, have been highlighted as:

• of special interest
•• of outstanding interest

## References

[1] Hamill S., Cota E., Chothia C., Clarke J. (2000). Conservation of folding and stability within a protein family: the tyrosine corner as an evolutionary cul-de-sac. J Mol Biol.

[2•] Nickson A.A., Clarke J. (2010). What lessons can be learned from studying the folding of homologous proteins?. Methods.

[3] Wetlaufer D.B. (1973). Nucleation, rapid folding, and globular intrachain regions in proteins. Proc Natl Acad Sci USA.

[4] Dolgikh D.A., Gilmanshin R.I., Brazhnikov E.V., Bychkova V.E., Semisotnov G.V., SYu V., Ptitsyn O.B. (1981). Alpha-Lactalbumin: compact state with fluctuating tertiary structure?. FEBS Lett.

[5] Karplus M., Weaver D.L. (1976). Protein-folding dynamics. Nature.

[6] Jackson S.E., Fersht A.R. (1991). Folding of chymotrypsin inhibitor 2. 1. Evidence for a two-state transition. Biochemistry.

[7] Itzhaki L.S., Otzen D.E., Fersht A.R. (1995). The structure of the transition state for folding of chymotrypsin inhibitor 2 analysed by protein engineering methods: evidence for a nucleation-condensation mechanism for protein folding. J Mol Biol.

[8] Paci E., Lindorff-Larsen K., Dobson C.M., Karplus M., Vendruscolo M. (2005). Transition state contact orders correlate with protein folding rates. J Mol Biol.

[9] Plaxco K.W., Simons K.T., Baker D. (1998). Contact order, transition state placement and the refolding rates of single domain proteins. J Mol Biol.

[10] Mayor U., Guydosh N.R., Johnson C.M., Grossmann J.G., Sato S., Jas G.S., Freund S.M.V., Alonso D.O.V., Daggett V., Fersht A.R. (2003). The complete folding pathway of a protein from nanoseconds to microseconds. Nature.

[11] Gianni S., Guydosh N.R., Khan F., Caldas T.D., Mayor U., White G.W.N., DeMarco M.L., Daggett V., Fersht A.R. (2003). Unifying features in protein-folding mechanisms. Proc Natl Acad Sci USA.

[12•] Banachewicz W., Religa T.L., Schaeffer R.D., Daggett V., Fersht A.R. (2011). Malleability of folding intermediates in the homeodomain superfamily. Proc Natl Acad Sci USA.

[13] Neuweiler H., Sharpe T.D., Rutherford T.J., Johnson C.M., Allen M.D., Ferguson N., Fersht A.R. (2009). The folding mechanism of BBL: plasticity of transition-state structure observed within an ultrafast folding protein family. J Mol Biol.

[14] Letarov A.V., Londer Y.Y., Boudko S.P., Mesyanzhinov V.V. (1999). The carboxy-terminal domain initiates trimerization of bacteriophage T4 fibritin. Biochemistry (Mosc).

[15] Panchenko A.R., Luthey-Schulten Z., Cole R., Wolynes P.G. (1997). The foldon universe: a survey of structural similarity and self-recognition of independently folding units. J Mol Biol.

[16] Maity H., Maity M., Krishna M.M.G., Mayne L., Englander S.W. (2005). Protein folding: the stepwise assembly of foldon units. Proc Natl Acad Sci USA.

[17] Hedberg L., Oliveberg M. (2004). Scattered Hammond plots reveal second level of site-specific information in protein folding: phi’ (beta++). Proc Natl Acad Sci USA.

[18] Lindberg M.O., Oliveberg M. (2007). Malleability of protein folding pathways: a simple reason for complex behaviour. Curr Opin Struct Biol.

[19] Lindberg M., Tångrot J., Oliveberg M. (2002). Complete change of the protein folding transition state upon circular permutation. Nat Struct Biol.

[20] Lindberg M.O., Haglund E., Hubner I.A., Shakhnovich E.I., Oliveberg M. (2006). Identification of the minimal protein-folding nucleus through loop-entropy perturbations. Proc Natl Acad Sci USA.

[21] Main E.R.G., Jackson S.E., Regan L. (2003). The folding and design of repeat proteins: reaching a consensus. Curr Opin Struct Biol.

[22•] Aksel T., Majumdar A., Barrick D. (2011). The contribution of entropy, enthalpy, and hydrophobic desolvation to cooperativity in repeat-protein folding. Structure.

[23] Lowe A.R., Itzhaki L.S. (2007). Biophysical characterisation of the small ankyrin repeat protein myotrophin. J Mol Biol.

[24] Lowe A.R., Itzhaki L.S. (2007). Rational redesign of the folding pathway of a modular protein. Proc Natl Acad Sci USA.

[25] Courtemanche N., Barrick D. (2008). The leucine-rich repeat domain of Internalin B folds along a polarized N-terminal pathway. Structure.

[26] Tripp K.W., Barrick D. (2008). Rerouting the folding pathway of the Notch ankyrin domain by reshaping the energy landscape. J Am Chem Soc.

[27] Ternström T., Mayor U., Akke M., Oliveberg M. (1999). From snapshot to movie: phi analysis of protein folding transition states taken one step further. Proc Natl Acad Sci USA.

[28] Shakhnovich E.I. (1998). Folding nucleus: specific or multiple? Insights from lattice models and experiments. Fold Des.

[29] Han J.-H., Batey S., Nickson A.A., Teichmann S.A., Clarke J. (2007). The folding and evolution of multidomain proteins. Nat Rev Mol Cell Biol.

[30] Hamill S., Steward A., Clarke J. (2000). The folding of an immunoglobulin-like Greek key protein is defined by a common-core nucleus and regions constrained by topology. J Mol Biol.

[31] Cota E., Steward A., Fowler S., Clarke J. (2001). The folding nucleus of a fibronectin type III domain is composed of core residues of the immunoglobulin-like fold. J Mol Biol.

[32] Geierhaas C.D., Paci E., Vendruscolo M., Clarke J. (2004). Comparison of the transition states for folding of two Ig-like proteins from different superfamilies. J Mol Biol.

[33] Fowler S., Clarke J. (2001). Mapping the folding pathway of an immunoglobulin domain: structural detail from phi value analysis and movement of the transition state. Structure.

[34] Lappalainen I., Hurley M.G., Clarke J. (2008). Plasticity within the obligatory folding nucleus of an immunoglobulin-like domain. J Mol Biol.

[35] Wright C.F., Lindorff-Larsen K., Randles L.G., Clarke J. (2003). Parallel protein-unfolding pathways revealed and mapped. Nat Struct Biol.

[36] Otzen D.E., Fersht A.R. (1998). Folding of circular and permuted chymotrypsin inhibitor 2: retention of the folding nucleus. Biochemistry.

[37] Neira J.L., Davis B., Ladurner A.G., Buckle A.M., Gay G.d.P., Fersht A.R. (1996). Towards the complete structural characterization of a protein folding pathway: the structures of the denatured, transition and native states for the association/folding of two complementary fragments of cleaved chymotrypsin inhibitor 2. Direct evidence for a nucleation-condensation mechanism. Fold Des.

[38] Sato S., Fersht A.R. (2007). Searching for multiple folding pathways of a nearly symmetrical protein: temperature dependent phi-value analysis of the B domain of protein A. J Mol Biol.

[39] Baxa M.C., Freed K.F., Sosnick T.R. (2008). Quantifying the structural requirements of the folding transition state of protein A and other systems. J Mol Biol.

[40] Nickson A.A., Stoll K.E., Clarke J. (2008). Folding of a LysM domain: entropy-enthalpy compensation in the transition state of an ideal two-state folder. J Mol Biol.

[41] Viguera A.R., Serrano L., Wilmanns M. (1996). Different folding transition states may result in the same native structure. Nat Struct Biol.

[42] McCallister E.L., Alm E., Baker D. (2000). Critical role of beta-hairpin formation in protein G folding. Nat Struct Biol.

[43] Nauli S., Kuhlman B., Baker D. (2001). Computer-based redesign of a protein folding pathway. Nat Struct Biol.

[44] Kim D.E., Fisher C., Baker D. (2000). A breakdown of symmetry in the folding transition state of protein L. J Mol Biol.

[45] White G.W.N., Gianni S., Grossmann J.G., Jemth P., Fersht A.R., Daggett V. (2005). Simulation and experiment conspire to reveal cryptic intermediates and a slide from the nucleation-condensation to framework mechanism of folding. J Mol Biol.

[46] Capaldi A.P., Kleanthous C., Radford S.E. (2002). Im7 folding mechanism: misfolding on a path to the native state. Nat Struct Biol.

[47] Friel C.T., Capaldi A.P., Radford S.E. (2003). Structural analysis of the rate-limiting transition states in the folding of Im7 and Im9: similarities and differences in the folding of homologous proteins. J Mol Biol.

[48] Friel C.T., Beddard G.S., Radford S.E. (2004). Switching two-state to three-state kinetics in the helical protein Im9 via the optimisation of stabilising non-native interactions by design. J Mol Biol.

[49•] Connell K.B., Miller E.J., Marqusee S. (2009). The folding trajectory of RNase H is dominated by its topology and not local stability: a protein engineering study of variants that fold via two-state and three-state mechanisms. J Mol Biol.

[50] Spudich G.M., Miller E.J., Marqusee S. (2004). Destabilization of the *Escherichia coli* RNase H kinetic intermediate: switching between a two-state and three-state folding mechanism. J Mol Biol.

[51] Dalessio P.M., Ropson I.J. (2000). beta-sheet proteins with nearly identical structures have different folding intermediates. Biochemistry.

[52] Dalessio P.M., Boyer J.A., McGettigan J.L., Ropson I.J. (2005). Swapping core residues in homologous proteins swaps folding mechanism. Biochemistry.

[53] Lorch M., Mason J.M., Clarke A.R., Parker M.J. (1999). Effects of core mutations on the folding of a beta-sheet protein: implications for backbone organization in the I-state. Biochemistry.

[54] Borgia A., Bonivento D., Travaglini-Allocatelli C., Di Matteo A., Brunori M. (2006). Unveiling a hidden folding intermediate in c-type cytochromes by protein engineering. J Biol Chem.

[55] Ivarsson Y., Travaglini-Allocatelli C., Morea V., Brunori M., Gianni S. (2008). The folding pathway of an engineered circularly permuted PDZ domain. Prot Eng Des Sel.

[56••] Haq S.R., Jürgens M.C., Chi C.N., Koh C.-S., Elfström L., Selmer M., Gianni S., Jemth P. (2010). The plastic energy landscape of protein folding: a triangular folding mechanism with an equilibrium intermediate for a small protein domain. J Biol Chem.

[57] Wildegger G., Kiefhaber T. (1997). Three-state model for lysozyme folding: triangular folding mechanism with an energetically trapped intermediate. J Mol Biol.

[58] Bollen Y.J.M., van Mierlo C.P.M. (2005). Protein topology affects the appearance of intermediates during the folding of proteins with a flavodoxin-like fold. Biophys Chem.

[59•] Hills R.D., Kathuria S.V., Wallace L.A., Day I.J., Brooks C.L., Matthews C.R. (2010). Topological frustration in beta alpha-repeat proteins: sequence diversity modulates the conserved folding mechanisms of alpha/beta/alpha sandwich proteins. J Mol Biol.

[60] Liu C.S., Gaspar J.A., Wong H.J., Meiering E.M. (2002). Conserved and nonconserved features of the folding pathway of hisactophilin, a beta-trefoil protein. Protein Sci.

[61] Chavez L.L., Gosavi S., Jennings P.A., Onuchic J.N. (2006). Multiple routes lead to the native state in the energy landscape of the beta-trefoil family. Proc Natl Acad Sci USA.

[62] Chen Y.-R., Clark A.C. (2006). Substitutions of prolines examine their role in kinetic trap formation of the caspase recruitment domain (CARD) of RICK. Protein Sci.

[63] Scott K.A., Randles L.G., Clarke J. (2004). The folding of spectrin domains II: phi-value analysis of R16. J Mol Biol.

[64] Scott K.A., Randles L.G., Moran S.J., Daggett V., Clarke J. (2006). The folding pathway of spectrin R17 from experiment and simulation: using experimentally validated MD simulations to characterize States hinted at by experiment. J Mol Biol.

[65] Wensley B.G., Gärtner M., Choo W.X., Batey S., Clarke J. (2009). Different members of a simple three-helix bundle protein family have very different folding rate constants and fold by different mechanisms. J Mol Biol.

[66••] Wensley B.G., Batey S., Bone F.A.C., Chan Z.M., Tumelty N.R., Steward A., Kwa L.G., Borgia A., Clarke J. (2010). Experimental evidence for a frustrated energy landscape in a three-helix-bundle protein family. Nature.

[67•] Wensley B.G., Kwa L.G., Shammas S.L., Rogers J.M., Clarke J. (2012). Protein folding: adding a nucleus to guide helix docking reduces landscape roughness. J Mol Biol.

[68] Borgia A., Hoffmann A., Pfeil S., Lipman E.A., Clarke J., Schuler B. (2012). Localizing internal friction along the reaction coordinate of protein folding by combining ensemble and single molecule fluorescence spectroscopy. Nat Commun.

[69] Wensley B.G., Kwa L.G., Shammas S.L., Rogers J.M., Browning S., Yang Z., Clarke J. (2012). Separating the effects of internal friction and transition state energy to explain the slow, frustrated folding of spectrin domains. Proc Natl Acad Sci USA.

[70] Mallam A.L., Jackson S.E. (2007). A comparison of the folding of two knotted proteins: YbeA and YibK. J Mol Biol.

[71•] Mallam A.L., Jackson S.E. (2012). Knot formation in newly translated proteins is spontaneous and accelerated by chaperonins. Nat Chem Biol.

[72••] Skrbić T., Micheletti C., Faccioli P. (2012). The role of non-native interactions in the folding of knotted proteins. PLoS Comp Biol.

[73] Dalal S., Balasubramanian S., Regan L. (1997). Protein alchemy: changing beta-sheet into alpha-helix. Nat Struct Biol.

[74] He Y., Chen Y., Alexander P., Bryan P.N., Orban J. (2008). NMR structures of two designed proteins with high sequence identity but different fold and function. Proc Natl Acad Sci USA.

[75] Scott K.A., Daggett V. (2007). Folding mechanisms of proteins with high sequence identity but different folds. Biochemistry.

[76••] Morrone A., McCully M.E., Bryan P.N., Brunori M., Daggett V., Gianni S., Travaglini-Allocatelli C. (2011). The denatured state dictates the topology of two proteins with almost identical sequence but different native structure and function. J Biol Chem.

[77•] Ratcliff K., Marqusee S. (2010). Identification of residual structure in the unfolded state of ribonuclease H1 from the moderately thermophilic Chlorobium tepidum: comparison with thermophilic and mesophilic homologues. Biochemistry.

[78] Kumar D., Chugh J., Sharma S., Hosur R.V. (2009). Conserved structural and dynamics features in the denatured states of drosophila SUMO, human SUMO and ubiquitin proteins: implications to sequence-folding paradigm. Proteins.

[79] Giri R., Morrone A., Travaglini-Allocatelli C., Jemth P., Brunori M., Gianni S. (2012). Folding pathways of proteins with increasing degree of sequence identities but different structure and function. Proc Natl Acad Sci USA.

[80] Jonsson A.L., Scott K.A., Daggett V. (2009). Dynameomics: a consensus view of the protein unfolding/folding transition state ensemble across a diverse set of protein folds. Biophys J.

[81] Best R.B., Fowler S.B., Herrera J.L.T., Steward A., Paci E., Clarke J. (2003). Mechanical unfolding of a titin Ig domain: structure of transition state revealed by combining atomic force microscopy, protein engineering and molecular dynamics simulations. J Mol Biol.

[82] Forman J.R., Yew Z.T., Qamar S., Sandford R.N., Paci E., Clarke J. (2009). Non-native interactions are critical for mechanical strength in PKD domains. Structure.

[83] Wolynes P.G. (2004). Latest folding game results: protein A barely frustrates computationalists. Proc Natl Acad Sci USA.

[84] Itoh K., Sasai M. (2006). Flexibly varying folding mechanism of a nearly symmetrical protein: B domain of protein A. Proc Natl Acad Sci USA.

[85] Haglund E., Lindberg M.O., Oliveberg M. (2008). Changes of protein folding pathways by circular permutation. Overlapping nuclei promote global cooperativity. J Biol Chem.

[86••] Koga N., Tatsumi-Koga R., Liu G., Xiao R., Acton T.B., Montelione G.T., Baker D. (2012). Principles for designing ideal protein structures. Nature.

[87] Steward A., McDowell G.S., Clarke J. (2009). Topology is the principal determinant in the folding of a complex all-alpha Greek key death domain from human FADD. J Mol Biol.

[88] Vendruscolo M., Paci E., Dobson C.M., Karplus M. (2001). Three key residues form a critical contact network in a protein folding transition state. Nature.

